# Are Metal Ions That Make up Orthodontic Alloys Cytotoxic, and Do They Induce Oxidative Stress in a Yeast Cell Model?

**DOI:** 10.3390/ijms21217993

**Published:** 2020-10-27

**Authors:** Vito Kovač, Borut Poljšak, Jasmina Primožič, Polona Jamnik

**Affiliations:** 1 Faculty of Health Sciences, University of Ljubljana, Zdravstvena pot 5, 1000 Ljubljana, Slovenia; vito.kovac@zf.uni-lj.si (V.K.); borut.poljsak@zf.uni-lj.si (B.P.); 2 Medical Faculty, University of Ljubljana, Vrazov trg 2, 1000 Ljubljana, Slovenia; jasminaprimozic@gmail.com; 3 Biotechnical Faculty, University of Ljubljana, Jamnikarjeva ulica 101, 1000 Ljubljana, Slovenia

**Keywords:** metal ion, orthodontic appliances, yeast, cytotoxicity, oxidative stress, lipid oxidation

## Abstract

Compositions of stainless steel, nickel-titanium, cobalt-chromium and β-titanium orthodontic alloys were simulated with mixtures of Fe, Ni, Cr, Co, Ti and Mo metal ions as potential oxidative stress-triggering agents. Wild-type yeast *Saccharomyces cerevisiae* and two mutants ΔSod1 and ΔCtt1 were used as model organisms to assess the cytotoxicity and oxidative stress occurrence. Metal mixtures at concentrations of 1, 10, 100 and 1000 µM were prepared out of metal chlorides and used to treat yeast cells for 24 h. Every simulated orthodontic alloy at 1000 µM was cytotoxic, and, in the case of cobalt-chromium alloy, even 100 µM was cytotoxic. Reactive oxygen species and oxidative damage were detected for stainless steel and both cobalt-chromium alloys at 1000 µM in wild-type yeast and 100 µM in the ΔSod1 and ΔCtt1 mutants. Simulated nickel-titanium and β-titanium alloy did not induce oxidative stress in any of the tested strains.

## 1. Introduction

Each part of an orthodontic appliance, whether an archwire, bracket or band, may differ from other parts by their composition and characteristics [1]. To make a fixed orthodontic appliance, some parts have to be joined together, whether that be by brazing, soldering or welding [2]. Many metallic alloys, ranging from different series of stainless steel, to nickel-titanium, pure titanium, and cobalt-chromium, are being used for manufacturing orthodontic devices, and all of them possess unique physical and mechanical properties [3]. Most of the orthodontic alloys form a so-called oxide layer, which makes them corrosion resistant. However, over time with exposure to the harsh, constantly changing oral environment, those biocompatible metal materials tend to locally corrode and biodegrade, thus releasing metal ions into the oral cavity [4,5]. In addition, galvanic corrosion might occur, when wire and brackets made out of two different alloys are soldered together, accelerating the release of metal ions [6].

The biocompatibility of orthodontic appliances has been extensively studied in the literature. In most cases, the release of metal ions from such appliances into artificial saliva either at low [5] or neutral pH [7,8,9] was analyzed, focusing on Ni, Cr, Fe and Co ions [10]. Although highly variable values of metal ion release were reported and the values did not exceed the recommended daily dietary intake [11], their toxic potential should not be neglected, especially on the local level within the oral cavity.

The ability of metal ions to generate reactive oxygen species (ROS) could contribute to the overall toxicity of fixed orthodontic appliances [12]. Iron, chromium, cobalt, nickel, titanium, and molybdenum are all classified as transition metals, which can undergo redox cycling reactions, thus forming ROS [13]. If the ever-increasing ROS molecules are not maintained at physiological levels, an overly large amount of generated ROS can lead to oxidative stress, which disrupts cellular redox homeostasis and consequently damages biomolecules (lipids, protein, and DNA). To counter these harmful effects and to scavenge overproduced ROS, a defense system comprising enzymatic and nonenzymatic systems is possessed by each cell. Superoxide dismutase (SOD), catalase (CAT) and glutathione peroxidase (GPx) are examples of some of the enzymes responsible for the maintenance of intracellular redox status [14].

Currently, in vitro cell lines are mostly being used to evaluate the cytotoxicity of orthodontic materials. To assess the agents’ ability to have toxic consequences on living cells [15], a yeast model organism *Saccharomyces cerevisiae* could also be used. Namely, its genome is well described [16], and the use of yeast as a model organism is relevant for human disease studies due to yeast proteins being homologous to human proteins [17]. In addition, features such as genetic tracing, ability to scale-up and the short generation time enable yeast to perform as an effective research model [18]. The similarity between *S. cerevisiae* and other eukaryotes in biochemical and molecular biological levels [19] appears to be useful when studying oxidative stress damage because of the potential mitochondrial respiration and oxidative damage accumulation in yeast [20].

To our knowledge, simulating orthodontic alloys with metal ions on this scale has not been previously performed. This study aimed to evaluate whether the metal ions that form selected orthodontic alloys are cytotoxic and capable of inducing oxidative stress in the yeast model organism *Saccharomyces cerevisiae*. Moreover, wild-type *S. cerevisiae* and two mutants lacking either SOD or CAT enabled a comparison to reveal whether superoxide anion and H_2_O_2_ lacking defense system contributes to metal-ion-induced toxicity.

## 2. Results

### 2.1. Cell Culturability

**Figure 1:**
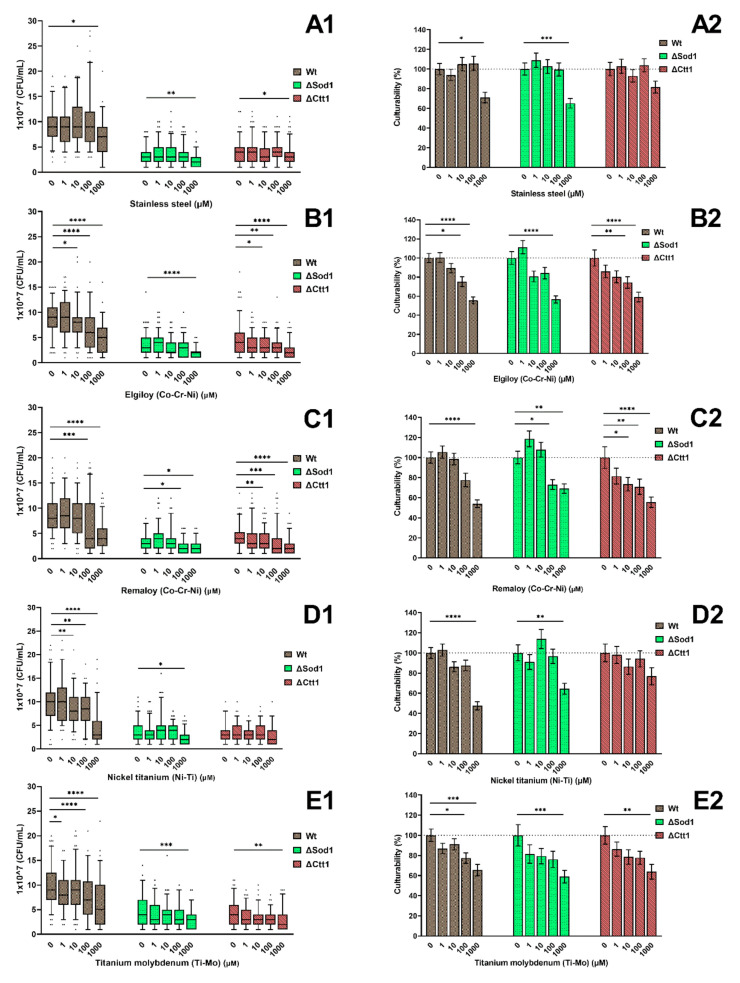
Cell culturability, expressed as CFU/mL, of yeast cells treated with different compositions and concentrations of metal ions. Wild-type yeast and two yeast mutants ΔSod1 and ΔCtt1 were subjected to metal ions simulating (**A**) stainless steel, (**B**,**C**) cobalt-chromium Elgiloy and Remaloy, (**D**) nickel-titanium, and (**E**) β-titanium alloys. The box-plots (**A1**–**E1**) show an absolute values of CFU/mL, while the grouped columns (**A2**–**E2**) present relative values according to the untreated control group. The untreated control group is set at 100%, and the dotted line represents 100% culturability. Significant differences (*p* < 0.05 are indicated with * (* *p* < 0.05, ** *p* < 0.01, *** *p* < 0.001, and **** *p* < 0.0001).

The toxic assessment of the treatment of each yeast strain (Wt, ΔSod1, ΔCtt1) with distinct metal ions combinations at different concentrations is presented in Figure 1 as CFU/mL cell counts (Figure 1A1–E1) and as percentages of cell culturability (Figure 1A2–E2). A lower cell culturability can be seen between the wild type and the other two mutants. All strains had a significantly lower cell culturability (*p* < 0.05) when treated with any of the metal ions at a 1000 µM concentration except for ΔCtt1 treated with Ni-Ti. Treatment with stainless steel (SS) (Figure 1A1,A2) at concentrations lower than 1000 µM had almost no effect on the cell culturability, while a significantly decreased percentage (*p* < 0.05) of cell culturability was detected when treating wild-type and ΔSod1 strains with concentrations of 1000 µM. The yeast strains treated with Elgiloy (ELG) (Figure 1B1) already had a significantly lower (*p* < 0.05) cell culturability at 10 µM concentrations for the wild-type and ΔCtt1 strains and 1000 µM concentration for the ΔSod1 strain compared with the untreated corresponding control strains. The mean culturability values for the cells treated with ELG (Figure 1B2) were significantly lower (*p* < 0.05) than those for the untreated corresponding controls for concentrations equal to or above 100 µM for wild type and ΔSod1; meanwhile, for ΔCtt1, a decrease in the cell survival rate was already detected, but not statistically significant, for the treatment with the 1 µM ELG concentration. Similarly, treatment with Remaloy (REM) at the 100 µM concentration and higher concentrations caused a statistically significant decrease (*p* < 0.05) in cell culturability of the wild-type and ΔSod1 strains; meanwhile, for the ΔCtt1 strains, REM concentrations of 10 µM significantly (*p* < 0.05) influenced cell culturability. A statistically significant decrease (*p* < 0.05) in cell culturability was detected for wild-type strains treated with 10 µM or higher concentrations of nickel-titanium (NiTi), whereas almost no effect on the culturability of ΔSod1 and ΔCtt1 was seen when treated with concentrations lower than 1000 µM. With the increasing concentration of β-titanium (TiMo), a decrease in culturability of every yeast strain was noticed and as in almost all other cases, the 1000 µM metal concentration had a statistically significant impact (*p* < 0.05) on the cell culturability.

### 2.2. Cell Metabolic Activity

**Figure 2:**
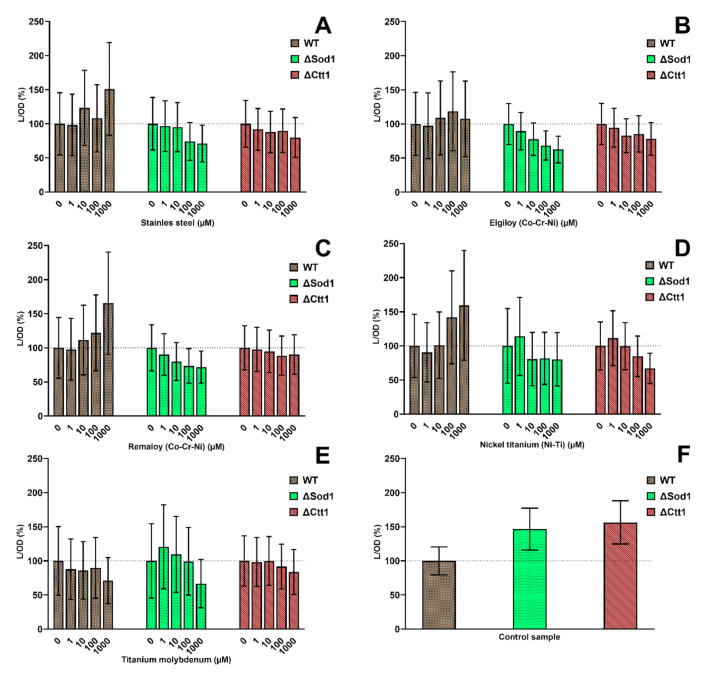
Metabolic activity of wild-type yeast and two yeast mutants ΔSod1 and ΔCtt1 treated with different combinations of metal ions: stainless steel (**A**), cobalt-chromium Elgiloy (**B**), Remaloy (**C**), nickel-titanium (**D**), and β-titanium (**E**). For each yeast strain, the untreated control group represents 100% (dotted line). A comparison among untreated control groups of each yeast strain is shown in the graph (**F**).

The metabolic activities of different yeast strains according to distinct metal ions combinations at different concentrations are presented in Figure 2. No statistically significant difference was seen when comparing the metabolic activity values for each treated sample with the corresponding untreated control. For SS, REM and NiTi, an increase in metabolic activity was concomitant with the increase in the concentrations used but only in the wild-type yeast strain. Treating wild-type yeast with ELG did not affect metabolic activity. On the other hand, treating wild-type yeast with TiMo had a minor decreasing effect of metabolic activity, and its effect gradually increased with the increasing concentrations of TiMo metal ion mixture. Mutant strains with different metal treatments displayed a decreasing trend in their metabolic activity with the increase in SS, ELG, REM and NiTi concentrations. For mutants treated with TiMo, only the highest concentration decreased their metabolic activity. A comparison between the untreated yeast strains showed a much greater metabolic activity of mutant yeast cells compared with wild-type yeast (Figure 2F).

### 2.3. Intracellular Oxidation

**Figure 3:**
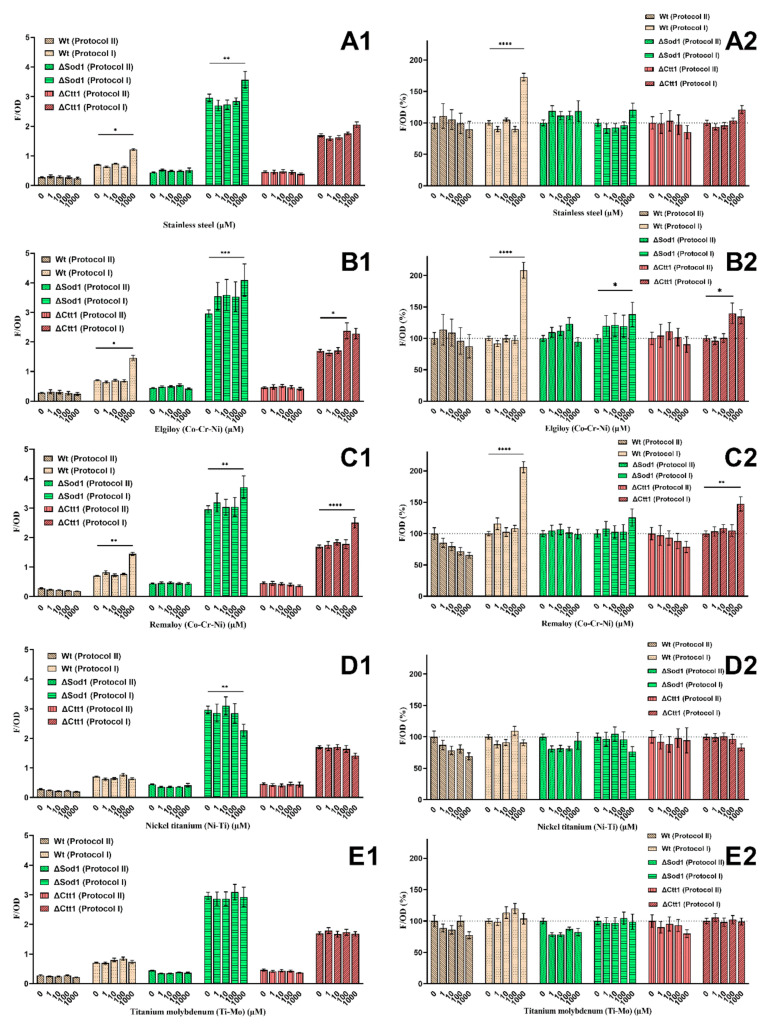
Intracellular oxidation performed with two different methods for wild-type yeast and two yeast mutants ΔSod1 and ΔCtt1, and metal treatment: stainless steel (**A**), cobalt-chromium Elgiloy (**B**) and Remaloy (**C**), nickel-titanium (**D**) and β-titanium (**E**). Grouped columns represent either absolute values (**A1**–**E1**) or relative values according to the untreated control group (**A2**–**E2**). For each yeast strain, the untreated control group represents 100% (dotted line). Significant differences (*p* < 0.05) are indicated with * (* *p* < 0.05, ** *p* < 0.01, *** *p* < 0.001, and **** *p* < 0.0001).

The ROS levels for the wild-type, ΔSod1, and ΔCtt1 yeast strains are shown in Figure 3 as F/OD (Figure 3A1–E1) and F/OD percentages (Figure 3A2–E2). Different ROS level measurements were obtained when the assessment was performed by adding fluorescent dye immediately after metal ion treatment (protocol I) or by adding it after 24 h (protocol II). Regardless of the metal ion combination and concentration, the values were twice as high for the wild-type strains, five-fold higher for ΔSod1 and three-fold higher for the ΔCtt1 strains when measured according to protocol II. According to the results obtained with protocol I, adding the fluorescent dye immediately after the 1000 µM metal ion treatment with SS, ELG and REM yielded significantly (*p* < 0.05) higher ROS values than the untreated control group in all yeast strains, but treating with the same and lower concentrations of TiMo had no significant effect on the ROS level. NiTi metal ion mixture at the 1000 µM concentration had the opposite effect on the ΔSod1 and ΔCtt1 mutant yeast cells. When detecting ROS levels, a significant decrease (*p* < 0.05) was measured for the ΔSod1 mutant, and a considerable decrease was measured for the ΔCtt1 mutant yeast.

According to the results obtained with protocol II, the addition of fluorescent dye after the 24 h metal ion treatment in many cases indicated a decrease in ROS levels, compared to the results from protocol I, with the exclusion of ΔSod1 mutant where stagnating values at SS, REM and TiMo metal treatment were observed. The wild-type strain exhibited a decrease in ROS level when increasing all metal ion mixtures, although not statistically proven. The NiTi and TiMo treatments had a similar effect on the yeast strains as SS, ELG and REM.

### 2.4. Oxidative Lipid Damage

**Figure 4:**
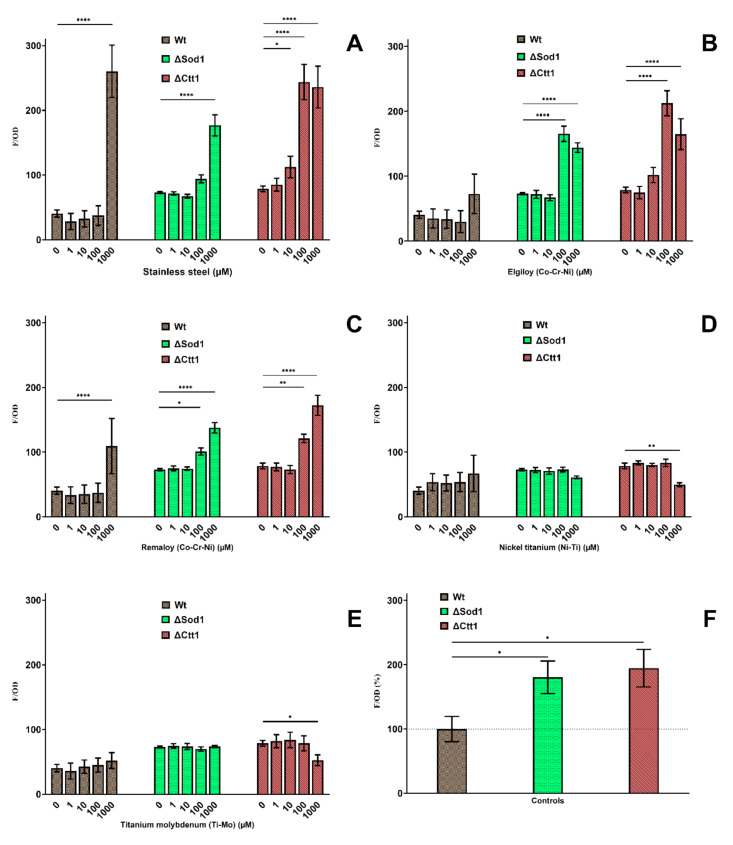
Influence of 24-h metal treatment on the formation of oxidative lipid damage. Wild-type, ΔSod1, and ΔCtt1 yeast strains were treated with different concentrations of stainless steel (**A**), cobalt-chromium Elgiloy (**B**) and Remaloy (**C**), nickel-titanium (**D**), and β-titanium (**E**). Graph (**F**) compares untreated control groups of different yeast strains relative to the wild type. Significant differences (*p* < 0.05) are indicated with * (* *p* < 0.05, ** *p* < 0.01 and **** *p* < 0.0001).

In Figure 4, a series of results are shown to evaluate whether the metal treatment caused lipid oxidative damage. SS, ELG, and REM showed a statistically significant increase (*p* < 0.05) in lipid oxidation in all yeast strains at the 1000 µM concentration compared with the untreated control, and for ΔSod1 and ΔCtt1 increased lipid oxidation occurred even at 100 µM. Treating wild-type yeast with NiTi and TiMo metal ions had no significant effect. Treating ΔSod1 with the same two metal ion mixtures had no proven effect, but a decrease in lipid oxidation in ΔCtt1 mutant can be seen at the 1000 µM concentration. The untreated mutant yeast cell lines ΔSod1 and ΔCtt had nearly two-fold higher levels of lipid oxidation compared with the untreated wild-type yeast (Figure 4F).

## 3. Discussion

Although several studies are available in the literature regarding the biocompatibility and potential cytotoxic impact of the metal ions released from implants [21,22,23], restorations [24,25,26] and orthodontic appliances such as Fe, Ni, Cr, Co, Ti, and Mo [27,28,29], no previous report has simulated orthodontic alloys with metal ion mixtures to evaluate their potential. Cultured animal or human cell lines such as fibroblasts [28] and osteoblasts [30] have been mainly used to evaluate the cytotoxic effect of orthodontic appliances, and currently, only a few studies [31,32] have included yeast for these type of studies. Limberger et al. [31] compared the results on *S. cerevisiae* with other cell lines and concluded that microorganisms are a reliable model for cytotoxic testing of orthodontic materials. Therefore, in the present study, *S. cerevisiae* was used as a model organism as it can provide a large number of results in a short time period due to its quick growth and easy handling. Simple and genetically well-annotated, yeast *S. cerevisiae* is the most well-understood eukaryotic organism and is often used to elaborate fundamental insights into mammalian cell biology. The major advantage of yeast-based studies lies in the high throughput that provides invaluable information about medical disorders related to metals. Possessing the same antioxidant defensive pathways as mammalian cells, yeast presents an appropriate model organism to investigate oxidative stress and related intracellular oxidative damage. Furthermore, the effect of combining some of the metal ions in the right ratios was evaluated to better simulate the composition of orthodontic alloys. Choosing to simulate stainless steel, nickel-titanium, β titanium, and two cobalt-chromium alloys with metal ions seemed to be appropriate as those five alloys are currently the most used, and their chemical compositions were previously reported by Arndt et al. [33] and Kusky [34].

Although parts of orthodontic appliances of different composition of metal alloys are available, it is the responsibility of the practitioner to ensure that the right kind of material is being used for the best possible patient treatment and safety. Being familiar with orthodontic alloys and their biocompatibility is an additional issue that should be considered when fulfilling patients’ needs or addressing patients’ problems. For instance, gingival hyperplasia, glossitis, erythema multiforme and labial desquamation might occur from metal ions that are released from orthodontic appliances [35]. Therefore, for a clinical procedure to be successful and safe, the biocompatibility of metal alloys, which combines fields of biology and engineering as well as patient risk factors and clinical experience, should concomitantly be considered [36]. Such improvement in clinical knowledge can be seen in the gradual abandonment of conventional brazing in favor of laser welding [37]. Not only are laser welded joints more durable than brazed joints, EDAX analysis clearly shows material corrosion of brazed joints, whereas laser welded joints had little to no corrosion [5]. That is why the biocompatibility of brazed joints is questionable, especially as the brazing solder used to combine alloys constitutes potentially dangerous metal ions such as zinc, copper and silver [38].

Almost all fixed orthodontic appliances are made out of metal alloys. Amongst them, stainless steel, nickel-titanium, β-titanium and cobalt-chromium alloys are the most commonly used orthodontic materials. Previous studies have shown that orthodontic appliances made out of the previously mentioned materials release metal ions over time [39]. The amount of released metal ions is largely dependent on the type of alloy, surrounding environment and the exposure time. While metal ions being non-biodegradable, they could accumulate in tissues and have toxic local and systemic effects [40,41]. The study was set to evaluate potential cytotoxic effects that fixed metal orthodontic appliances might have through the excretion of metal ions. One approach would be submerging orthodontic materials in medium, enabling a direct contact with the cells. Another approach includes the orthodontic material being incubated in some sort of medium (e.g., artificial saliva) for a long period of time and the emerged metal ion eluate is being used to treat the cells. But by using constituent elements, like metal chlorides, a controlled environment can be simulated, where all the metal ions that compromise an orthodontic alloy are released in the same manner, thus enabling the necessary information about the potential cytotoxic damage, that might occur during the orthodontic treatment. The different methodological approaches used in both in vitro and in vivo studies of metal ion release and inconsistent results [42] made it difficult to select correct metal ion concentrations. In some in vitro experiments, cumulated concentrations of metal release over 30 days of some orthodontics alloys could not be detected or could be as much 7000 ng/mL [43]. Metal ion concentrations of in vivo experiments involving patients’ saliva had a much lower release, between 4 and 30 ng/mL [10]. Locally in vivo, near to the orthodontic alloy, metal ion concentration could be much higher than in the saliva. To investigate possible adverse effects and mechanism of action as well as to find the lowest observed adverse effect level [44] of selected cytotoxic or oxidative stress parameters, the metal concentration interval between 1 and 1000 µM was used in the presented study.

It was shown that metal ions might impact cell culturability. One of the reasons is the causation of oxidative stress. All metal ions used in the study are transition metals, which can catalyze Fenton and Haber–Weiss reactions, resulting in ROS formation, such as superoxide anions (•O^−^_2_), hydrogen peroxide (H_2_O_2_), and hydroxyl radicals (•OH) [45]. Each organism (cell) contains various antioxidant defense systems, including SOD and CAT, which are two enzymes that provide a defense grid for removing ROS [46]. The yeast mutants lacking defensive enzymes, ΔSod1 and ΔCtt1, exhibited higher ROS formation and consequently more oxidative damage. A superoxide anion (•O^−^_2_) is formed in the respiratory chain of every mitochondria-containing cell; although less reactive than other ROS, it represents a precursor for most of them [47]. Cytosolic SOD (Sod1) scavenges for •O^−^_2_ and converts it to H_2_O_2_, which is further degraded to H_2_O and O_2_ by cytosolic catalase (Ctt1) [48]. In our study, yeast mutants lacking the Sod1 gene have higher ROS levels than the Ctt1-lacking mutants despite the same amount of lipid oxidation. Meanwhile, SOD is the only antioxidant enzyme capable of converting •O^−^_2_, and H_2_O_2_ can be reduced by both catalases and glutathione peroxidases. Importantly, there were more isozymes of both SOD and catalase enzyme in the cell [46]. That is why the mutants in our study, lacking one form of the enzyme, can survive and adapt to the oxidative environment. Limberger et al. [31] used multiple yeast mutants with either antioxidant defense or DNA repair deficiency to assess the cytotoxicity of orthodontic materials but did not demonstrate any oxidative stress-related ROS formation or oxidative damage. Nevertheless, yeast has proven to be a suitable model organism, as we were able to detect oxidative stress and an impact on viability at 10× lower concentrations when oxidative defense-deprived strains were used. Using a wild-type strain as well as the ΔSod1 and ΔCtt1 mutants, we could compare the occurrence of oxidative stress among them.

A major difference among yeast strains can be seen in the cell culturability values. Untreated mutated yeast cells had at least two-fold lower cell culturability than untreated wild-type yeast. This may be because mutants lacking genes for antioxidant defense tend to adapt slowly to new metal-induced stress environments. When comparing metal-ion treated cells with the untreated control, the treated mutants had lower cell culturability values than the untreated control. Any metal ion concentration of 1000 µM had a significant negative effect on yeast survival. Even the 100 µM concentration of all metal ion combinations, except stainless steel, showed a negative effect. Meldawar et al. [49] observed that at approximately 425 µM of pure Ni ions in human epithelial embryotic cell lines (L132) resulted in 50% viability, but Ti ions and the combination of the two had no impact on the cell lines even at 3750 µM concentrations. The results cannot be compared with our study because an LC50 was obtained at the 1000 µM NiTi concentration, and TiMo alloy comprising almost strictly Ti ions showed a dose-dependent decrease in cellular viability. Issa et al. [50] used metal chlorides to assess the survivability of human gingival fibroblast (HGF) and oligodendrocytes (MO3.13). The cobalt concentrations were the first to cause 50% survivability, followed by the nickel and then chromium concentrations. The LC50 for HGF was Co = 705 µM, Ni = 828 µM and Cr = 1971 µM; and for MO3.13, it was Co = 215 µM, Ni = 817 µM and Cr = 2084 µM. Although they did not test combinations of different metal ions, the obtained information is valuable as it seems that choosing the right model organism to evaluate cytotoxicity is as important as the metal ion composition of the alloy. Namely, Terpilowska et al. [51] showed that a combination of metal ions can have a synergistic or even antagonistic effect that cannot be observed when treating cells with a single metal.

Assessing the metabolic activity of cells yielded very disparate results. Specifically, the assay quantifies the ATP present in cells, which is proportional to the number of viable, metabolically active cells in the medium. This might not be the case with wild-type yeast strain because its cell culturability and metabolic activity do not share the same outcome when treated with some of the metal ions. In stress situations, ATP depletion is the step before viability loss [52], because the membrane and electron transport chain are first affected by toxic metals [53]. Under mild stress conditions, cells might use ATP to activate defense systems. On the other hand, under increased oxidative stress, which results in intracellular oxidative damage, ATP is decreased due to the damage to mitochondria [54]. Akhova et al. [55] explained that ATP increase could occur due to oxidative stress response, such as antioxidant genes being transcripted [56]. Meanwhile, unnecessary energy-consuming processes are canceled. Because the mutants in our study lack one of the most important genes for oxidative stress defense, they cannot cope as well with oxidative stress as the wild-type strain. Notably, the untreated mutant strains had higher metabolic activity compared with the untreated wild-type strain; hence, activation of the antioxidant defense system was already established.

The intracellular oxidation assay provided information on whether the cytotoxicity of metal ions is due to ROS formation. The measured values after 24 h of incubation of yeast cells with metal ions showed that only the 1000 µM concentrations of SS, ELG, and REM metal ion increased ROS levels but only when administering the fluorescent dye as soon as the metal treatment starts. The results can be deceiving for two reasons. First, if the obtained fluorescence is plotted against the number of culturable cells, the values are much higher compared with the untreated control sample, especially for mutant strains, as their viability decreased in a dose-dependent manner. H_2_DCF-DA dye can cross the cell membrane, where it is cleaved by intracellular esterase and further oxidized by ROS to exhibit high fluorescence [57]. This process can be conducted only in live cells. A problem of dye leakage could occur [58], but it was excluded by cell washing before measurement. Second, the fluorescent values for untreated yeast mutants were higher than for untreated wild-type yeast. Thus, the mutants already had to cope with the emerging ROS from the start and for 24 h until the analysis was performed. That is, they had ample time to adapt and overcome the stress or succumb to it. That could explain why no increase in ROS was observed when intracellular oxidation after 24 h of treatment was measured.

To further investigate oxidative stress, malondialdehyde (MDA), a product of lipid oxidation, was analyzed. As in ROS detection, the lipid oxidation in mutants was almost two times higher when compared with the wild type. An identical series of values were obtained when comparing the amount of MDA to the amount of ROS, which corresponds to our findings. The MDA levels for the wild type were significantly different for SS, ELG, and REM at 1000 µM and for both mutants even at 100 µM. Indeed, neither NiTi nor TiMo induced ROS formation or lipid oxidation, despite previous studies showing that Ni [59], Ti [60], and Mo [61] ions have that ability. A small increase in lipid oxidation could be seen in the wild type, but it was not significant.

The metal concentrations of Cr, Fe, Mo and Ni ions at 1000 µM and higher have been proven to decrease cell viability and the occurrence of apoptosis [62]. Caicedo et al. [63] tested the harmful effects of metal ions from medical devices on Jurkat cells and provided a scale for them, and the negative effects were as follows: Ni > Co > Mo > Cr ≥ Fe. Interestingly, the two regarded as most harmful, Ni and Co, had different modes of action. Lower concentrations of Ni were needed to induce oxidative stress-related DNA damage and apoptosis (50 and 100 µM, respectively) compared with Co, but the Co concentration could decrease viability and inhibit proliferation at 500 and 100 µM, respectively.

## 4. Materials and Methods

### 4.1. Preparation of Yeast Cultures

Stock culture of wild type *S. cerevisiae* ATCC 204508 (American Type Culture Collection, Manassas, WV, USA), ΔSod1 *S. cerevisiae* Y06913 (EUROSCARF, Oberursel, Germany) and ΔCtt1 *S. cerevisiae* Y04718 (EUROSCARF, Oberursel, Germany) were grown in yeast extract-peptone-dextrose broth (Merck, Darmstadt, Germany) and incubated in an incubator shaker (InforsHT, Bottmingen, Switzerland) at 28 °C and 220 RPM. The genotypes of each yeast strains are listed in Table 1. The early stationary phase of *S. cerevisiae* yeast strains was adjusted to approximately 1 × 10^7^ cell/mL. The yeast strains were then kept in PBS medium (Merck, Darmstadt, Germany) in the desired stationary phase until metal ion treatment.

### 4.2. Preparation of Metal Ion Solutions

As described in Table 2, 0.2 M metal ion stock solutions of 5 orthodontic alloy types were prepared. High-purity salts of FeCl_3_ × 6H_2_O, CrCl_3_ × 6H_2_O, NiCl_2_ × 6H_2_O and CoCl_2_×6H_2_O (all bought from Merck, Darmstadt, Germany) as well as TraceCERT**^®^** titanium and molybdenum standards (Merck, Darmstadt, Germany) were dissolved with sterile ddH_2_O and had their pH adjusted to 7. Before treatment, metal stock solutions were briefly sonicated in an ultrasound bath (Sonis 3 GT, Iskra Pio, Šentjernej, Slovenia).

### 4.3. Metal Treatment of Yeast Strains

Five metal ion mixtures with final concentrations of 1, 10, 100 and 1000 μM were used to treat yeast strains with a final volume of 10 mL. One sample of each yeast strain was not treated with metal mixtures as it served as a control. The incubation period of 24 h followed in an incubator shaker (InforsHT, Bottmingen, Switzerland) at 28 °C and 220 RPM.

### 4.4. Cell Culturability

After 24 h, metal-treated cells and the control sample of untreated cells were diluted with PBS medium in the range from 10^−2^ to 10^−5^. Ten microliters of the metal-treated yeast solution was spotted on a solid YPD-agar plates (Merck, Darmstadt, Germany) and incubated for 48 h at 28 °C. After the incubation, formed colonies were counted and the culturability results were expressed as 1 × 10^7^ colony-forming units per milliliter (CFU/mL).

### 4.5. Cell Metabolic Activity

For the assessment of cell metabolic activity, BacTiter-Glo™ Microbial Cell Viability Assay (Promega, San Luis Obispo, CA, USA) was used and performed according to the manufacturer’s instructions. In brief, a volume of the provided reagent was mixed with the same amount of cell suspension in a 96-well plate; after 5 min of incubation, the formed luminescence and optical density at 650 nm (OD650) were recorded using a Tecan microplate reader (Männedorf, Switzerland). The results were expressed as a ratio of luminescence/optical density (L/OD) relative to the untreated control.

### 4.6. Intracellular Oxidation

Fluorescent dye 2′,7′-dichlorofluorescein diacetate (H_2_DCF-DA) [65,66] with two slightly different approaches was used to detect intracellular reactive oxygen species (ROS). In the first approach (protocol I), immediately after the addition of metal ion mixtures to the yeast suspension, H_2_DCF-DA (Merck, Darmstadt, Germany) was added to a final concentration of 10 µM. After 24 h of incubation, the cells were washed once with PBS medium, and fluorescence (excitation/emission = 488/520 nm) and OD (650 nm) were measured using a Varioskan™ LUX (Thermofisher, Waltham, MA, USA) microplate reader. In the second approach (protocol II), 24 h metal-treated and untreated cells were first washed once with PBS medium, and then the dye was added in a final concentration of 10 µM. After 30 min of incubation in the dark, the cells were washed again with PBS medium, and then fluorescence and OD with the same parameters were measured using Varioskan™ LUX (Thermofisher, Waltham, MA, USA). In both cases, the results were expressed as F/OD relative to the untreated control.

### 4.7. Oxidative Lipid Damage

For the analysis of oxidative lipid damage [67], the thiobarbituric acid reactive substances (TBARS) assay was employed. It is based on the detection of malondialdehyde (MDA), a marker for oxidative stress damage, which emits fluorescent light at 555 nm. Treated and untreated cells were washed once with PBS medium after 24 h metal ion mixtures treatment. A reagent containing 91.8 mM trichloroacetic acid (Merck, Darmstadt, Germany), 2.5 mM thiobarbituric acid (Merck, Darmstadt, Germany), 45.4 μM butylhydroxytoluene (Merck, Darmstadt, Germany) and 25 mM HCl (Merck, Darmstadt, Germany) was added to the pellet, which was thoroughly resuspended and homogenized twice (Bullet Blender Storm 24, Next Advance, Troy, New York, NY, USA) with 5 min of incubation on ice in between. The homogenate was incubated at 90 °C (Thermomixer R, Eppendorf, Hamburg, Germany) for 30 min followed by 10 min of incubation on ice. Butanol (Merck, Darmstadt, Germany) was added to the homogenate and centrifuged at 10,000× *g* for 10 min. Fluorescence (excitation/emission = 515/555 nm) and OD (650 nm) were measured on a Varioskan™ LUX (Thermofisher, Waltham, MA, USA) microplate reader. The results were expressed as F/OD relative to the untreated control.

### 4.8. Statistical Analysis

For CFU/mL counts, ten technical repetitions were performed for each of the seven biological repetitions. When testing the cell metabolic activity, five biological repetitions with two technical repetitions were used for analysis. H_2_DCF-DA was applied immediately after the metal treatment on six biological replicates with two technical replicates each. To measure the intracellular oxidation after 24 h, at least three biological replicates with two technical replicates were used. The assessment of the lipid oxidation occurrence was performed with five biological replicates and two technical replicates. For the analysis, the untreated control sample of every yeast strain was set at 100%, and the treated samples were plotted against it. Visual presentation and statistical analysis were performed with GraphPad Prism (version 8.02 for Windows, GraphPad Software, La Jolla, CA, USA, www.graphpad.com). Shapiro–Wilk and D’Agostino and Pearson tests were used to analyze the normal distribution of the acquired data. Normally distributed data were analyzed with one-way ANOVA followed by Dunnett’s post hoc test for multiple comparisons and non-normally distributed data were analyzed with Kruskal–Wallis test followed by Dunn’s post hoc test. Cutoff for the statistical significance of data was considered when *p* < 0.05.

## 5. Conclusions

The metal ion concentrations of 1000 µM were proven to be cytotoxic to *S. cerevisiae*, even the 100 µM concentrations in the case of simulated cobalt-chromium treatment. The same concentrations of simulated stainless steel and cobalt-chromium alloys exhibited ROS formation, which was observed in yeast mutants ΔSod1 and ΔCtt1 already at 100 µM. Untreated yeast mutants had higher ROS and MDA basal levels than the untreated wild-type strain, indicating that even under untreated conditions, both mutants had increased intracellular ROS levels due to incomplete defense. When evaluating titanium-containing nickel-titanium and β-titanium ion mixtures, there was no evidence of oxidative stress formation, indicating that a different kind of cytotoxic mechanism must exist when yeast cells are exposed to these two simulated alloys.

According to the presented results, the metal ions released from fixed orthodontic appliances into the oral cavity due to corrosion and biodegradation possess a low-risk profile for users, as only extremely high metal concentrations induced cytotoxicity and oxidative stress, as presented in our in vitro study on the *S. cerevisiae* model organism; however, increased ROS might occur on the local level in the oral cavity, especially in patients with a deficiency in antioxidant defense systems, which should be further investigated.

## Figures and Tables

**Table 1:** *Saccharomyces cerevisiae* strains used in the study.

Strain	Genotype	Source
ATCC 204508 (Wt)	MATa; SUC2; mal; mel; gal2; CUP1; flo1; flo8-1; hap1	American Type Culture Collection, Manassas, Virginia, United States
Y06913 (ΔSod1)	BY4741; MATa; ura3Δ0; leu2Δ0; his3Δ1; met15Δ0; YJR104c::kanMX4	EUROSCARF, Oberursel, Germany
Y04718 (ΔCtt1)	BY4741; MATa; ura3Δ0; leu2Δ0; his3Δ1; met15Δ0; YGR088w::kanMX4	EUROSCARF, Oberursel, Germany

**Table 2:** Metal ion ratios for orthodontic alloys adapted from the manufacturer’s (Dentaurum, Ispringen, Germany) material safety data sheet [64].

	Metal Composition (*w*/*v*)					
Orthodontic Alloy	Fe	Ni	Cr	Co	Ti	Mo
Stainless steel (SS)	72%	10%	18%			
Cobalt-chromium (Elgiloy—ELG)	18%	15%	20%	40%		7%
Cobalt-chromium (Remaloy—REM)	5%	21%	20%	50%		4%
Nickel-titanium (NiTi)		55%			45%	
β-titanium (TiMo)					78%	12%

## Abbreviations

ROS	Reactive oxygen species
SOD	Superoxide dismutase
CAT	Catalase
GPx	Glutathione peroxidase
L	Luminescence
OD	Optical density
H_2_DCF-DA	2′,7′-dichlorofluorescein diacetate
MDA	Malondialdehyde
CFU	Colony forming units
PBS	Phosphate-Buffered Saline
Wt	Wild type
SS	Stainless steel
ELG	Elgiloy
REM	Remaloy
NiTi	Nickel-titanium
TiMo	Β-titanium
EDAX	Energy-dispersive X-ray spectroscopy
